# Analyzing the “Opposite” Approach in Additions to Historic Buildings Using Visual Attention Tools: Dresden Military History Museum Case

**DOI:** 10.3390/jemr19010007

**Published:** 2026-01-12

**Authors:** Nuray Özkaraca Özalp, Hicran Hanım Halaç, Mehmet Fatih Özalp, Fikret Bademci

**Affiliations:** 1 Department of Architecture, Faculty of Fine Arts and Design, Siirt University, 56000 Siirt, Turkey; nuray.ozkaraca@siirt.edu.tr; 2 Department of Architecture, Faculty of Architecture, Eskisehir Technical University, 26555 Eskişehir, Turkey; hhhalac@eskisehir.edu.tr; 3 Independent Researcher, 81620 Düzce, Turkey; fozalp79@gmail.com; 4 School of Architecture, University of Liverpool, Liverpool L69 7ZG, UK

**Keywords:** eye tracking, visual attention, contemporary extensions, historical buildings

## Abstract

From past to present, modern additions have continued to transform historic environments. While some argue that contemporary extensions disrupt the integrity of historic buildings, others suggest that the contrast between past and present creates a meaningful architectural dialog. This debate raises a key question: in contrasting compositions, which architectural elements draw more visual attention, the historic or the modern? To address this, a visual attention-based analytical approach is adopted. In this study, eye-tracking-based visual attention analysis is used to examine how viewers perceive the relationship between historical and contemporary architectural elements. Instead of conventional laboratory-based eye-tracking, artificial intelligence-supported visual attention software developed from eye-tracking datasets is employed. Four tools—3M-VAS, EyeQuant, Attention Insight, and Expoze—were used to generate heat maps, gaze sequence maps, hotspots, focus maps, attention distribution diagrams, and saliency predictions. These visualizations enabled both a qualitative and quantitative comparison of viewer focus. The case study is the Military History Museum in Dresden, Germany, known for its widely debated contemporary addition representing an oppositional design approach. The results illustrate which architectural components are visually prioritized, offering insight into how contrasting architectural languages are cognitively perceived in historic settings.

## 1. Introduction

From the past to the present, modern additions have been made to historic environments. These additions can enrich the historic setting and impart a symbolic character to the structures being expanded [1]. While some consider such interventions to be incongruous, others regard them as a successful blend of old and new, creating a balanced contrast. In this context, the debate centers on which architectural elements attract more visual attention in contrasting approaches: historic or contemporary. This highlights the need for a method to both visually analyze and analytically evaluate such additions. In this study, the chosen method is visual tracking/visual attention. Eye tracking, also known as eye monitoring or visual attention, is the process of tracking the movement of the eyes to determine exactly where a person is looking and for how long. Research shows that it takes only 3–5 s to capture and sustain human attention. The eye is unconsciously activated during the first 3–5 s of pre-attentive gaze to evaluate a visual stimulus by perceiving visual details, configurations, and symmetries [2]. Eye tracking, a method that measures where people look and for how long, allows researchers to see through the eyes of users and gain insight into visual attention [3]. This method was initially widely used in fields such as medicine, education, entertainment, neuromarketing, and psychology [2]. Later, it was included in architectural and urban design research fields [4]. To date, a wide range of eye-tracking and visual attention studies have been conducted [5,6]. In recent years, the development of artificial intelligence has enabled rapid and non-participatory visual attention tests based on AI-driven prediction models. AI-assisted predictive eye tracking has become a biometric tool that is beginning to find application in research on our interaction with the built environment [7].

In this study, examples of the “contrasting” approach, which is the most controversial of the new design approaches in the historic environment, were analyzed with the visual attention method. For visual attention, 3M-VAS, EyeQuant, Attention Insight, and Expoze software, which are artificial intelligence applications developed using experimental data obtained from eye tracking, were used, and the latest versions available through the official websites of these software tools were employed. Diagrams and visual analyses from the software are presented to explain which architecture is more engaging and why. There are two main objectives of this research. The first aim of the research is to determine the suitability of the contrast method, which is one of the most controversial new building approaches in the world of conservation, on the existing historic building and at the same time to give a quantitative answer to the question of “what kind of contrast effect is achieved” with the visual attention method. Secondly, our aim is to introduce 3M-VAS [8], Attention Insight [9], EyeQuant [10], and Expoze [11] visual attention software, which are frequently used in academic studies, and to examine their effectiveness comparatively. For these purposes, the study is based on the following two main hypotheses:

**H1:**  *Historical elements systematically generate more pre-attentive focus than contemporary architectural additions.*

**H2:**  *Although different AI-based visual attention analysis tools produce largely consistent results, a certain level of representational differences is inevitable.*

## 2. Theoretical Framework

### 2.1. Eye-Tracking and Visual Attention

Eye tracking may seem modern, but its origins date back to the 19th century [12]. Initially, researchers studied eye movements based on observations without measuring equipment. A mirror was placed on the pages of the book being read by the subject, and the experimenter standing behind the subject observed the movements of the subject’s eyes through this mirror. Although this method was quite primitive, it still yielded some interesting results regarding visual perception during reading. There are some inaccuracies about who was the first to observe eye movements during reading. Most sources state that the first researcher to describe eye movement during reading was the French ophthalmologist Louis Émile Javal in the late 1870s, which is also considered to be the beginning of the era of eye-tracking studies. In 1879, Javal concluded that reading does not require the eyes to move continuously along a line of text, as previously assumed. He explained that reading is not a linear process, meaning that the subject’s eyes do not move continuously as they pass over each line, but rather a series of rapid movements (skipping) and short pauses (fixations) [13].

The study of eye movements was first conducted by E. Huey 1898 [14]. In 1908, Edmund Huey 1908 [14] developed the initial eye-tracking device, which required participants to wear a contact lens-like device to monitor their pupils and was considered uncomfortable. In 1948, Hartridge and Thompson 1948 [14] invented the head-mounted eye-tracking device, which allowed for greater freedom of movement [14]. Russian psychologist Alfred Lukyanovich Yarbus conducted significant studies in the 1950s and 1960s that laid the foundation for eye-tracking technology, showing that eye movements are linked to the user’s attention and focus on the task [13]. By the 1980s, biometric eye-tracking technology had advanced as devices became more accurate and less intrusive. In 2001, Tobii 2001 [15] made a significant contribution to visual attention computing by introducing the first remote eye-tracking device. Tobii, one of the world’s leading companies in eye tracking, develops systems that measure head and eye movements with high accuracy using camera and near-infrared-based sensor technologies. These systems utilize advanced signal processing and artificial intelligence methods to determine gaze point and analyze attention allocation [15,16]. While Tobii devices are frequently cited in the literature as a primary tool for providing real-time eye movement data, this study did not utilize a hardware-based eye tracking system; analyses were conducted solely using AI-based visual attention estimation software. With computers now able to process real-time visual tracking data, eye-tracking technology has been utilized to analyze users’ responses [17]. Recently, with the advancement of artificial intelligence technologies, visual attention tests can be conducted without participants, and eye-tracking systems have become more efficient with AI-supported prediction technologies. Over 150 years of development, eye-tracking technology has progressed from observational studies to intrusive tools, then to less intrusive devices, and finally to non-intrusive systems based on AI-powered predictive technology. AI-powered predictive eye tracking represents a significant advancement over traditional eye-tracking methods. For commercial applications that require quick results, this AI-assisted prediction technology offers valuable and dependable outcomes. In this study, visual attention software such as 3M-VAS, Attention Insight, EyeQuant, and Expoze are utilized to simulate eye-tracking applications with artificial intelligence.

### 2.2. Visual Attention and Architecture

According to research, it takes only 3–5 s to capture and sustain human attention. The eye is unconsciously activated during the first 3–5 s of pre-attentive gaze to evaluate a design by perceiving visual details, configurations, and symmetries [2]. What do observers see when looking at a particular image/landscape? Where is their attention drawn unconsciously during the pre-attentional stage (which occurs automatically without any effort or attention on the part of the perceiver, and during this stage the object being looked at is discriminated according to its basic characteristics (such as color, texture, shape, etc.))? [18]. This question is one of the fundamental questions designers and architects must solve. To draw the viewers’ attention to the structure, the architect needs to know the elements and factors that play a role in attracting attention [19]. Furthermore, if there is consistency in the visual attention of different individuals, designers can organize the design by emphasizing certain mechanisms to meet the needs of users [20]. Pre-attention-oriented eye-tracking studies reveal that attention is constantly drawn to the presence of people and especially to the human face, for example, every human presence in the image of a building or street scene is quickly detected. Another finding is that our gaze tends to focus on by details, contrasts, and structures that make general geometric sense [2]. In addition, factors such as changing the color of some details, changing the time of day and year, different scale, quantity, or brightness of architectural detail can also change the perspective from which people interpret architecture [21]. Biophilic effects such as vegetation, clouds, etc., have also been found to attract a certain amount of pre-attentive gaze due to their fractal lines and distract attention from the building of interest [22].

According to the theory of Cognitive Architecture—in other words, Neuroarchitecture—there are architectural principles that people subconsciously prefer. Traditional buildings are accepted to attract more subconscious attention than modern ones [23]. Research that aims to elucidate prior attention and cognitive responses to buildings can guide designers to designs that yield more analytical results. The neuro-architecture approach has also been integrated into another emerging technology, virtual reality [24]. By creating a virtual representation of different proposed buildings, architects can quickly (and relatively inexpensively) test reactions and strive to produce the best design with more objective findings rather than relying on more subjective opinions. Design pilots reveal findings that can have a major impact on how buildings are designed, from how empty facades are treated to how buildings are designed [25]. In this context, the issue of additions to historical buildings, which is one of the most debated issues in the field of conservation, and the effect of these additions on the historical structure were examined in the context of “visual attention”.

### 2.3. New Building Approaches in Historical Environment

The form/shape, material, construction technique, and style of new designs in historic neighborhoods are among the most debated issues in the field of conservation. Although there are some recommendations on how new designs should be, there are no clear rules regarding design parameters such as the scale (height and width), form, mass, orientation, and proportion of a new addition. In this regard, design approaches (new buildings and additions) in historic environments are classified into two types: the compatible approach (imitation/repetition and interpretation) and the contrasting/opposite approach [26]. Design approaches that develop between these two extremes, being compatible with the features of the historical texture while differentiating from the features of the historical building to which the addition is made, are shaped by design concepts (location, scale, proportion, material, color, texture, etc.). The elements that contribute to the visual integrity of a contemporary construction added to a historical building are listed below.

*Location*: Inconsistencies among the facade elements of historic buildings can potentially harm the image of a historical area [27]. The placement of a new addition to a historic building should preserve, rather than obscure or damage, the view of the building’s important facades [28]. The location of the new addition should be planned to minimize obstruction of the monumental facades. The visual negative impact of an addition on a historic building can be reduced by placing it on a side that is less obstructive to the view of the main facade. Using this approach, the additional structure is positioned near secondary or non-primary facades or behind the monument [29].

*Scale*: The scale of new buildings can be classified into three types according to their relationship with historic buildings: large-scale, harmonious-scale, and small-scale. Koolhaas and Mau 1994 [30] defined large-scale architecture as the ultimate point of architecture and mentioned that such buildings break the rules by exceeding the scale of the city and human perception. They argued that large-scale buildings reject context and are devoid of scale, architectural composition, tradition, transparency, ethics, and even urban fabric. In contrast, a harmonized scale involves designing buildings according to the volumetric dimensions of the surrounding historic context, such as height, width, and length [30]. Moreover, to reduce the visual impact of a new addition, it is desirable to make it the same height (compatible scale) or shorter (small scale) than the main historic building [31].

*Form*: In conservation theories, form is one of the features that reflect the esthetic value of a historic building. Form also plays a significant role in maintaining the visual integrity of buildings and shaping their architectural character. When conservation theories from 1968 to 2011 are analyzed with regard to form, it is evident that design approaches focusing on the form of historic buildings are emphasized in most conservation theories [30]. The form of new buildings in a historic context should be compatible with the dominant form of nearby buildings. The form of the new building does not need to imitate the neighboring building, but it should be appropriately in harmony with it. In form-finding, it is crucial to establish a connection between the new building and the historic context [32]. It may not be possible to distinguish the old building from the new if the newly added structure has a shape that is repeated from the historic part and is unrecognizable. While the form of the new building should be compatible with the historic building and preserve its character, it should also be easily distinguishable from the historic building. In this way, the site’s historical development can be more clearly understood, and the new building does not compromise the visual identity of the historic structure. However, for the new addition to be the center of attention, it should not be so different, distinct, and conspicuous in the eyes of the public. One of the most appropriate methods to meet global guidelines is using abstract forms. The abstraction approach tries to extract the essence and nature of historical buildings and transform them into a specific form. It explicitly represents hidden meaning, complex ideas, and classical concepts [29].

*Material and Color*: The proposed materials do not have to resemble precisely the materials of the historic building, but should be compatible with the monuments [33]. On the other hand, it should not be so prominent that it stands out from the historic monument and directs the gaze towards it. For example, using glass cladding material involves two different attitudes. In some cases, glass can attract too much attention due to its unwanted reflection in the historical context. In other cases, it is used to reflect the image of the surrounding historical buildings and to promote the historical place. In this way, its presence is neutralized. In addition, glass provides visual continuity with its transparency and reduces the negative visual impact in the area. Using new materials consistent in texture and composition and compatible with ancient materials is also an appropriate method. For example, constructing the facade of a new building in exposed concrete next to a historic stone building can create an engaging and visually appealing contrast. This approach adds a modern touch to the new building while preserving the historic character and visibility of the old building [29].

The subject of this study is the contrasting approach method, which is one of the approaches of additional building in the historic environment. It method is based on the contrast of combining the historic environment with new buildings that is perceived as a kind of protest of the new time against the old and therefore arouses much interest as the most controversial and problematic method in the progression of architecture. Architectural environment always reflects the values of the era and society in which it was created, and always coexists with the artificial or natural environment before its creation. Buildings built in the past exist now. Buildings constructed in our time will exist in the future. Buildings built in our time will exist in the future. Because of this duality of the temporal and social nature of architecture, contrast in different features is inevitable [34]. Since architectural heritage is culturally diverse, the approval of contrasting views should be considered as one of the core values of a heritage building [35]. There are many examples of contrasting designs in the historic environment/building worldwide [36]. This study examines the Dresden Military History Museum, whose new addition was designed by Daniel Libeskind, as a case study.

## 3. Materials and Methods

In the study, the contrast approach, the most controversial of the new building design approaches in the historical building, is the subject of analysis. In this context, the Military History Museum in Dresden, Germany, was selected as a case study to predict the observers’ reactions to the visuals and was analyzed using the visual attention method. Artificial intelligence software tools—3M-VAS, EyeQuant, Attention Insight, and Expoze—developed using experimental data from eye-tracking studies, were used to analyze visual attention. Diagrams and visual analysis obtained from eye attention software explain which architecture is more interesting and why.

### 3.1. AI-Based Visual Attention Tools

Modern technologies allow for almost completely non-intrusive recording of human gaze at presented objects [21]. In recent years, AI-based predictive eye-tracking tools developed from extensive eye-tracking experimental data are becoming increasingly available and widely used. In this way, researchers have started conducting fast and participant-free visual attention tests. In this study, 3M-VAS, EyeQuant, Expoze, and Attention Insight visual attention software, which academic studies commonly use, were used and analyzed comparatively. One key objective of this study is to introduce and examine eye-tracking tools based on artificial intelligence prediction technology, emphasizing their usability in architectural and urban design research. Applications based on AI-assisted prediction technology have been designed by different companies using numerous actual eye-tracking records and trained by obtaining a high level of correlation between real eye-tracking studies and their simulation. Users can access the software through web pages (Table 1). The image uploaded for analysis is scanned fully automatically within a few seconds, and the results are then available for download as a report.

**Figure 1:**
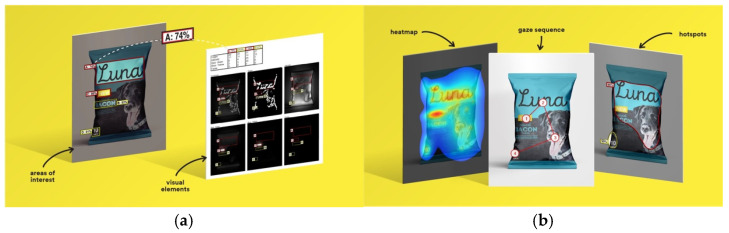
3M-VAS visual attention analysis: (**a**) Heat Map, Gaze Sequence and Hotspots; (**b**) areas of interest and visual elements [8].

3M VAS: 3M™ Visual Attention Software (VAS) is an artificial intelligence tool that utilizes a wealth of experimental eye-tracking data from over 30 years [37]. VAS instantly predicts what users will see at first glance (within the first 3–5 s) with 92% accuracy and provides a report including Heat Map, Hotspots, Gaze Sequence, and Visual Elements. Heat Map: provides an easy-to-see overview of the salient areas of the design. Hotspots: scores the likelihood of attracting the viewer’s attention in the hotspot regions of the Heat Map. Order of View: shows the predicted sequence in which the user notices the top 4 areas. It also provides probability scores for the likelihood of seeing the areas of interest specified in the design. Visual Elements: Basic design principles and color theory show that people are consistently attracted to the same visual elements. Accordingly, VAS simulates how the human brain instinctively responds to certain visual elements, such as edges, density, red/green color contrast, blue/yellow color contrast, and surfaces (Figure 1) [8].

**Figure 2:**
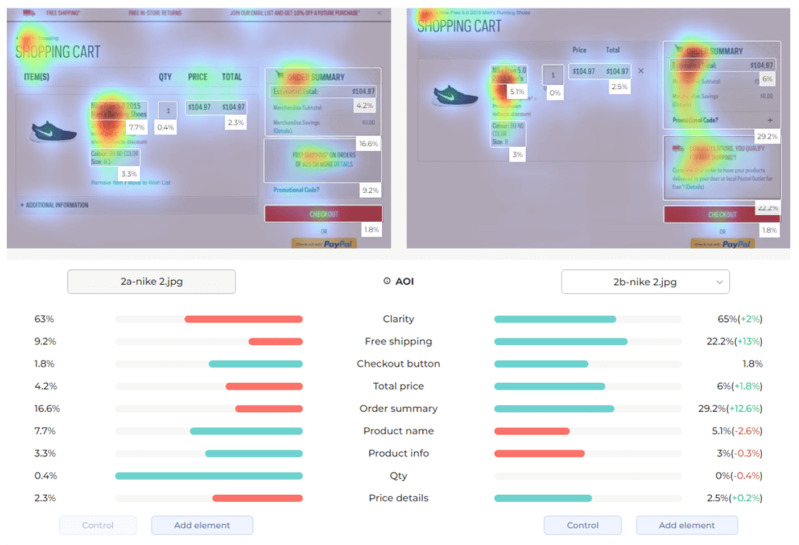
Predictive eye tracking with Attention Insight [9].

Attention Insight: It is a software that can generate eye tracking reports (attention heat maps, attention percentage, focus map, clarity score, contrast map) with an up to 96% accuracy rate without any human participant with the support of artificial intelligence. The Heat Map visually represents the most salient areas using warm and cool colors. For example, the most salient fields receive warmer colors like red and yellow. Attention Percentage: allows you to define an AOI (area of interest) and then see how much attention it receives. Focus Map: shows which parts of the design are noticed or overlooked within the first 4 s. Focus Map: allows users to see the most critical elements of designs. In addition, A/B tests allow for design comparisons, making it easy to observe differences in attention percentage and clarity scores through changes in the heat map and focus map (Figure 2) [9].

**Figure 3:**
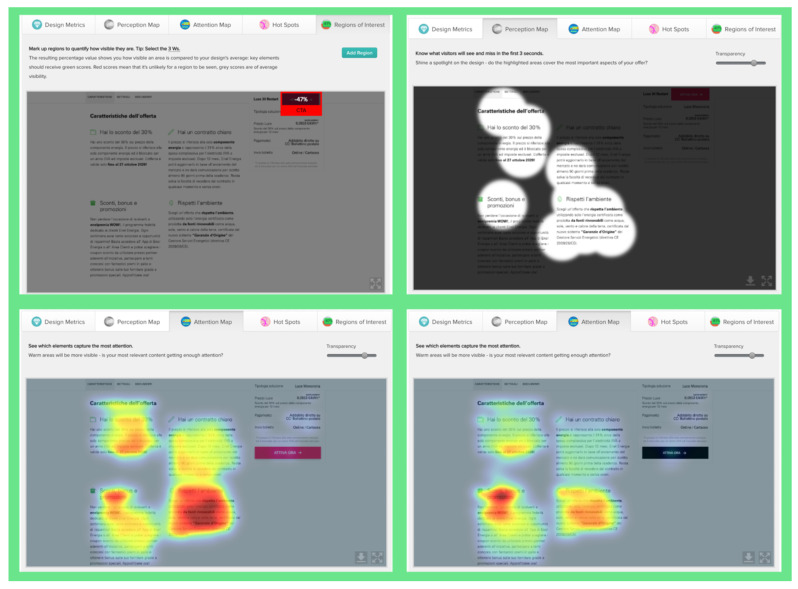
Perceptual map representation with EyeQuant and visual clarity and excitingness scores [10].

EyeQuant: This software leverages neuroscientific models and AI simulations to generate visual attention maps by predicting eye-tracking data. These maps show which visual elements users focus on and which areas attract more attention. EyeQuant can generate various outputs, including the Perception Map, Attention Heat Map, Hot Spots, Clarity Map, and Excitement Map. The Perception Map highlights the content that will be seen (or missed) in the first 3 s. It shows whether your most important or relevant content will be seen instantly by tracking variables that affect attention, such as contrast, color, brightness, edges, faces, and size. The attention heatmap shows which of your content receives the most attention. Warmer (red) areas have the most visibility. It estimates fixation volume (most seen), not fixation duration (attention length). With Hot Spots, users can identify the ten most visually prominent points within the analyzed design. The larger the circle, the more visible the focal point. At the same time, Hot Spots allows you to establish a visual hierarchy that aligns the most visible focal points with your design objectives. The clarity map measures how clean and clear your design is. The Clarity Map measures how clean and clear your design is. It provides a clarity score on a scale from 0 to 100 (with 50 representing the average) and visually indicates potential clutter within the design. A score of 0 indicates an overly cluttered and busy design, while a score of 100 signifies a highly intelligible, well-organized, or minimal design. The Excitement Map shows how exciting and stimulating the design looks. A score between 0 and 100 is given (where 50 represents the average). A score of 0 indicates an overly calm or boring design, while 100 indicates a highly stimulating one (Figure 3) [10].

**Figure 4:**
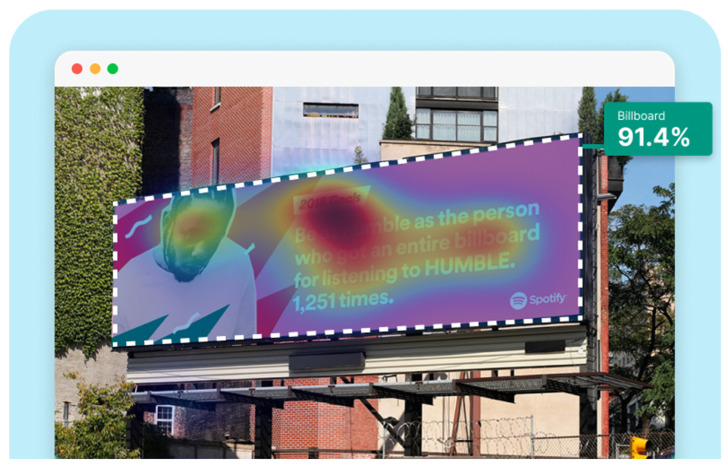
Example of heat map and attention percentage of interest with Expoze [11].

Expoze: Predicts visual attention using an algorithm trained with traditional eye-tracking data. The software generates a heatmap for each uploaded image, allowing you to identify the elements that attract attention. The heatmap can then be analyzed by adding Areas of Interest. It assesses how well significant elements in an image attract attention. For example, it can accurately predict how well a brand logo or a product attracts attention in an advertisement (Figure 4) [11].

**Figure 5:**
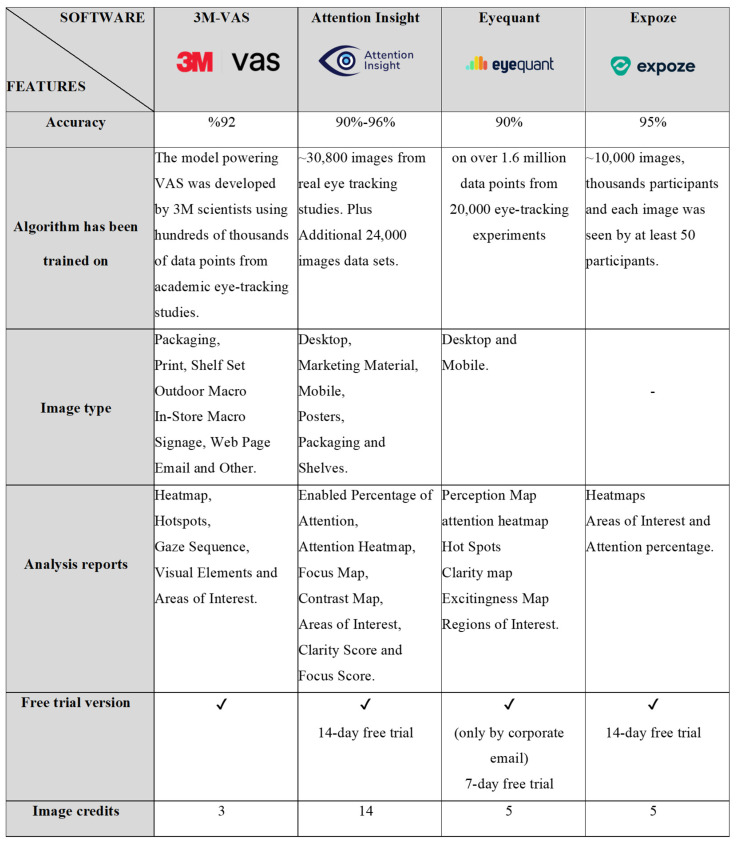
Comparison of visual attention software based on artificial intelligence prediction technology.

Applying the eye tracking/visual attention method in architectural research plays a significant role in understanding how users perceive designed spaces and evaluating the effectiveness of architectural designs. Artificial intelligence-supported visual attention software such as 3M VAS, Attention Insight, Eyequant, and Expoze can offer different perspectives and features for design analysis in the field of architecture. Although each software can be used to identify the visual focal points of users, understand spatial interactions, and improve design processes, it is noteworthy that 3M’s VAS is mostly used in scientific studies in the architectural context. The reason for this is the presence of the Environmental Designer identity as a role when creating membership in 3M-VAS, and the presence of the outdoor option in the image type option to be analyzed [2,4,22,38,39,40,41] (Figure 5).

### 3.2. Case Study: Military History Museum (Dresden, Germany)

This article aims to examine the effects of additions/new buildings constructed with an opposing approach in historical buildings, using the visual attention method. In line with this purpose, the Military History Museum in Dresden, Germany, which includes an additional building designed with an opposite approach, was subject to examination.

**Figure 6:**
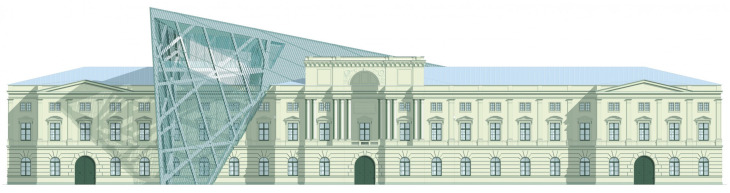
Military History Museum, Germany by Architect Daniel Libeskind [45].

The Dresden Military History Museum, the official museum of the German Armed Forces, has assumed different and contradictory identities throughout history [36]. First built as an armory in 1876, the building became a museum in 1897 through an official conversion. However, in 1989, a decision was made to close it to the public [42]. In 2001, Daniel Libeskind won the international competition organized to redesign the building, and his design sharply disrupted the classical symmetry of the original structure. The massive 14,500-ton, five-storey wedge of glass, concrete, and steel interrupted the classical layout of the former arsenal and added a modern facade that emerged from a traditional historic look. During World War II, the wedge faces the area where the bombardment of Dresden began and offers an influential view [36,42,43]. With this addition, the facade of the 19th-century museum was to include 20,000 square meters of galleries that highlight the themes of violence, war, and human nature [44]. The historic building’s central entrance portal is the focal point of the facade and the most ornate aspect of the composition. While the symmetrical and regularly planned old building houses the traditional chronological exhibition, Libeskind’s wedge-shaped addition contains the thematic exhibition that explores the social and human origins of violent conflict [43] (Figure 6).

### 3.3. Visual Data Preparation

Three images were used as stimuli during the study. Two of these three images were daytime photographs of the facade of the Military History Museum, while the third was a nighttime photograph. The aim here was to determine the change in attention in the daytime views before and after the addition, as well as to determine the state of attention under the effect of nighttime lighting after the addition. The daytime views of the historical building before the addition (unmodified historical building) and after the addition were taken from the work of Marta Rusnak and Mateusz Ratajczak [46]. In these images, the daytime photographs were modified—the clouds were removed and replaced with a clear blue gradient. The clouds could have distorted the results of the experiment due to their complex and highly dynamic structures. The third image analyzed is the nighttime view of the historic building after the addition and has been scaled to match the first two images (Table 2). Clouds are not visible in the nighttime photograph; the background is flat and unremarkable. The images have been processed so that the final versions are the same size, ratio, resolution, and composition. Thanks to all these preparations, all three facades could be compared as they did not contain any elements that could distract attention from the main subject of the analysis. Apart from this, no color correction or other processing was required for the images. The areas of interest were determined by the authors and were attempted to be defined in such a way that the extension structure would be completely covered in a separate drawing in the post-extension state of the historic building. The location of the extension on the facade encouraged the marking of three separate areas of interest: the facade of the extension, the right facade after the extension, and the left facade after the extension. The location of the extensions on the historical facades (such as centered on the historical facade or located on the edges of the historical facade) may increase or decrease the number of areas of interest mentioned. While the 3M-VAS and Expoze tools allow areas of interest to be marked as polygons in freehand drawing format, the Attention Insight and EyeQuant tools allow them to be marked as rectangles. Considering the diversity of contemporary additional geometries, this makes the 3M-VAS and Expoze tools more operational.

### 3.4. Limitations of the Study

In this study, limitations include ignoring 3D perception, focusing on visual experience by ignoring sensory experience, the accuracy rates of the software used, limitations of the software due to the use of a DEMO version, limitations due to the selection of images to be analyzed, and constraints due to the number of cases in testing the research question.

Ignoring the Perception of Three Dimensions: In this study, visual attention is analyzed using 2D images. While visual information remains fixed in two-dimensional visuals, the perceptual experience in a three-dimensional environment is far more dynamic and limitless [47]. As such, the analyses conducted in this study can only capture a portion of the actual visual perception. Because the images are static, the role of movement in perception is not considered in visual attention analyses based on 2D content. Therefore, results derived from 2D images may not fully represent real-world experiences. In a 3D context, movement and depth significantly influence perception and must be accounted for. Although this is beyond the scope of the current study, future research should consider using video-based analyses to better reflect real-life visual engagement.

Focusing on the visual experience while ignoring the sensory experience: Interaction with urban space is shaped not only by sight but also by many sensory experiences (sight, hearing, smell, taste, and touch) [40]. In this study, as in most visual attention studies, the focus is on visual experience, ignoring other senses. However, interaction with urban space is not limited to visual perception. Various sensory experiences are also significant. Therefore, solely focusing on visual experiences is a limitation when evaluating the overall interaction with urban space.

Accuracy rates of software: The accuracy and reliability of the software used in visual attention analysis are significant. The visual attention analyses conducted in the study were tested based on snapshots and the accuracy rate of the relevant software (92%). However, factors that may affect the accuracy of these analyses (e.g., changes in light and color balance, etc.) should be considered. Photos with a size of less than 600 pixels can also affect the accuracy of the results [8]. Therefore, in the election of the photographs analyzed in the study, attention was paid to light and color balance, and images with a resolution higher than 600 pixels were used.

Limitations of the software due to the use of the DEMO version: The free trial of the respective software allows you to analyze a limited number of images before purchasing a subscription by creating a membership. Therefore, to view more than three images, it is necessary to buy a subscription or to process through a new member. In this study, four member accounts were used to generate free visual attention analyses. In future studies, it is recommended to have a subscription to increase the number of cases to be analyzed or to perform visual attention analysis on more members.

Limitations related to the choice of images to be analyzed: The choice of which image to use in the analysis of relevant cases may add bias to the study. For example, close-ups, distant shots, photographs taken at different times/seasons, under different weather conditions, etc., many factors may affect the findings [48]. For this reason, visual attention analyses were repeated using different photographs of the cases selected in the study.

Limitations due to the number of cases in testing the research question: The research question, “Which architectural elements attract more attention when considering new additions and new building effects in historic areas: historic elements or modern elements”? The testing of this research question is limited to the case analyzed within the scope of the study.

## 4. Results and Comparison

This section presents the findings of the visual attention analysis of the examined historical building. The visual attention tests were conducted on different images of the Military History Museum using artificial intelligence-supported visual attention software.

Visual attention visualizations obtained through 3M-VAS, using daytime and night-time images of the Military History Museum before and after the construction of the additional, are presented in Table 2. The goal was to determine whether the historical building or the additional building attracted more attention. The software offers options for analyzing different types of images, including Print (Packaging, Print, Shelf Set), Environment (Outdoor Macro, In-Store Macro, Signage), and Digital (Web Page, Email, and Other). For this study, the Environment and Outdoor Macro options were selected as the most appropriate for the images being analyzed. The results of the visual attention analysis, including the heat map, hot zones, gaze sequences, areas of interest, and visual elements, are presented in Table 2.

The heat map shows areas where observers are most likely to look in red, areas where attention decreases in blue, and areas where no attention occurs in black. In the daytime view, the historical facades of the Military History Museum attracted more attention than the additional building, while in the night view, attention was drawn to the additional building due to lighting and glass surfaces.

Hot spots are simplified versions of the heat map results, indicating elements most likely to be seen. In the daytime view, attention was generated at a rate of 42%, 48%, and 56% before the addition, and 51%, 52%, and 81% after the additional. This suggests that the additional increased visual attention on the historic building.

Areas of interest, marked as rectangles and polygons, show that the additional structure attracted 30% of attention in the daytime view and 40% at night.

Gaze sequences reveal the positions most frequently observed by observers, and at the Military History Museum, all sequences focus on historical facades.

Visual elements simulate how the human brain is naturally drawn to certain elements in the Military History Museum, such as edges, density, color contrasts, and faces. Analytically, all analyses with 3M-VAS show that historical elements attract more attention than modern elements in the Military History Museum (Table 3).

Visual attention visualizations obtained through Attention Insight, using daytime and night-time images of the Military History Museum before and after the construction of the additional, are presented in Table 4. The full analysis output (Attention Heatmap, Focus Map, Contrast Map, AOIs, Clarity Score, and Focus Score) from the visual attention results generated by Attention Insight is presented in Table 3. This software offers various options for the type of image to be analyzed, including Desktop, Marketing Material, Mobile, Posters, Packaging, and Shelves. In this study, with guidance from Mindaugas (R&D Manager, Customer Success Manager, and Copywriter at Attention Insight), the Marketing Material option was selected as the most general and most suitable model for the analysis. This model is designed, among other purposes, to analyze content such as real estate advertisements, which often include images of buildings. The model has been trained accordingly. It is also noted that the Clarity Score and Contrast Map are not meaningful for the visual attention analysis of buildings. On the other hand, the Attention Heatmap, Attention Percentage, Focus Map, and Focus Score are considered significant and valid indicators.

The Attention Heatmap shows which elements attract attention. It reveals the most dominant focal point—the part that stands out the most. Also, you can see the distribution of attention—is it concentrated or scattered? When analyzing the attention heatmap of the Military History Museum, it is observed that in the daytime view after the construction of the additional, all the areas where red and its shades are concentrated correspond to the historical facades, suggesting that the additional does not attract significant attention. In the nighttime view, however, the lighting effect causes the viewer’s attention to be partially drawn to the additional building.

The Focus Map highlights the areas that users notice in the first few seconds and hides those that are ignored. On this map, regions with high brightness levels attract more attention, while darker areas draw less. When examining the focal map of the Military History Museum, it is evident that the contemporary addition appears almost completely dark in the daytime view following the construction of the additional structure. This suggests that the additional has low visibility in terms of visual attention. The centrally located entrance facade, with its classical architecture, stands out as the brightest area. This section becomes a visual focal point due to its high contrast and symmetrical layout. These findings indicate that the traditional facade is more dominant in terms of visual attention, whereas the new additional receives relatively little attention. In the nighttime view, however, the lighting effect causes the viewer’s focus to shift partially toward the additional.

Focus score measures the level of attention and concentration in your image. In images with several attention-grabbing elements, attention is divided among them, reducing the visibility of individual parts. This can make the image difficult to comprehend or interpret. A higher score means more concentrated attention. While the focus score of the Military History Museum before the additional building was 93 out of 100, the daytime focus score after the additional building was 92. This result shows that the concentration of attention is largely preserved after the additional, with a decrease in only one point. The historic building still receives intense visual attention.

Attention areas of interest (AOIs) can quantitatively measure how attention-grabbing the marked fields are. Attention Insight areas of interest can only be drawn as rectangles, and drawing alternatives are limited. When the facade of the historic building where the additional structure was added was marked as an area of interest, the attention percentage before the additional structure was 43, while the attention percentage after the additional structure was 29. In this case, the additional building decreased the attention of the facade to which it was added by 14 percent, and the percentage of attention to historical facades increased. In the night view of the additional, the attention percentage rose to 37%, a change attributed to the effect of lighting.

Visual attention visualizations obtained through EyeQuant, using daytime and night-time images of the Military History Museum before and after the construction of the additional, are presented in Table 4. This software offers Desktop and Mobile options for analyzing different types of images. In this particular study, the image type selected for analysis in the software was Desktop. The comprehensive analysis output, including the Attention Heatmap, Perception Map, Hot Spots, Clarity Map, Excitingness Map, and Regions of Interest, from the visual attention results obtained with EyeQuant is presented in Table 5.

The attention heatmap shows which of your content receives the most attention. Warmer (red) areas have the most visibility. It predicts fixation volume (most seen), not fixation duration (attention length). When the attention heat map of the Military History Museum is examined, in the daytime view after the additional building, it is seen that all the places where red and its tones are concentrated correspond to historical facades, and it can be said that the additional building does not attract attention. In the night view, it is understood that most of the attention is drawn to the additional building due to the lighting effect.

The Perception Map highlights the content that users will see (or miss) in the first 3 s. It tracks the variables that affect attention, such as contrast, color, brightness, edges, faces, and size, to show if your most important or relevant content will be seen instantly. When the focus map of the Military History Museum is analyzed, it is seen that in the daytime view after the additional, the contemporary additional is almost completely hidden in darkness. This situation shows that the additional has low visibility in terms of visual attention. The entrance facade with classical architecture located in the center of the historical building draws attention as the brightest area. It is seen that this area has become a focal point with its high contrast and symmetrical layout. In the night view, it is understood that most of the focus is drawn on the additional structure due to the lighting effect. With these findings, although it can be said that the traditional facade is more dominant in terms of visual attention and the new additional building remains in the background, lighting designs appear as a significant element that directs attention in night views.

Hot Spots represent the 10 most attention-grabbing areas in your design, with the size of the circle indicating the prominence of the focal point. When analyzing the hot spots of the Military History Museum, it is evident that in the daytime view after the additional, the circles are concentrated on the historical facades, with the additional not attracting much attention. In the night view, the circles are concentrated on the additional building due to the effect of lighting.

The Clarity map assesses the cleanliness and clarity of your design, providing a score out of 100. A score of 50 represents the average page on the internet, with 0 indicating extreme clutter and 100 representing a very clean design. When examining the Clarity map of the Military History Museum, green areas are present throughout the building, resulting in an increased clarity score from 92 to 94, a 2-point increase.

The Excitingness Map evaluates how exciting and stimulating your design appears, with a score ranging from 0 to 100. A score of 0 indicates a calm or boring design, while 100 signifies an extremely stimulating design. Red areas on the map represent calming elements, and targeting these areas can enhance visual stimulation. Upon analyzing the Excitement Map of the Military History Museum, red areas were observed on the additional structure, increasing the overall excitement score of the historical building from 71 to 77, a 6-point increase.

Areas of Interest (AOIs) are used to quantify the significance of marked areas with quantitative scores. EyeQuant areas of interest are represented as rectangles, with limited drawing alternatives. When marking the facade of the historic building with the additional structure as the area of interest, the attention percentage decreased from 21 to 14 after the addition. This indicates that the additional structure reduced attention to the facade by 7 percent, while increasing attention to the historic facades. In the night view of the additional building, the attention percentage was measured at 48, with the increase in attention attributed to lighting.

Visual attention visualizations obtained through Expoze, using daytime and night-time images of the Military History Museum before and after the construction of the additional, are presented in Table 5. The complete analysis output (Heatmap, AOIs, and Attention percentage) of the visual attention results performed with Expoze is given in Table 6. The absence of an option to select the type of image to be analyzed in this software can be considered a disadvantage. However, Expoze offers an advantage by allowing areas of interest to be drawn using rectangles, circles, triangles, and polygons.

When analyzing the Expoze heatmap results, it is observed that red and its shades are concentrated on the facades of the historical building, indicating that these areas attract more attention than the addition. In the night view heatmap after the addition, red tones also appear on the additional building due to lighting effects. Despite this, while the attention percentage increased from 21.9% to 32.6%, the historical building still attracts more attention than the addition.

Table 7 and Table 8 present and compare the heatmaps generated by 3M-VAS, EyeQuant, Attention Insight, and Expoze for the daytime and nighttime views of the Military History Museum, before and after the addition was constructed. These heatmaps comparatively show how different attention estimation algorithms determine the perceptual attention points of architectural structures. It is observed that there are some general trends as well as some differences in the generated attention maps. General trends: all attention estimation models focus on the salient geometric and esthetic elements of buildings. Attention is generally directed to areas of high contrast, detailed architectural elements, and prominent lighting. As for differences in attention distribution, for example, Expoze and EyeQuant outputs predict a much higher proportion of red hotspots than VAS and Attention Insight. Similarly, VAS and EyeQuant seem to be more prone to report fragmentation of attention with much smaller values than the other tools. When looking at different heatmaps from the same source image, there is some very general agreement and a real consensus that the historic structure attracts more attention than the additional structure. These findings support the hypothesis that, although different AI-based visual attention analysis tools produce largely consistent results, a certain level of representational differences is inevitable.

It is noteworthy that the areas identified by VAS, Attention Insight, and Expoze as hotspots likely to attract attention are not perceived in the same way by EyeQuant. EyeQuant, for whatever reason, seems to focus much more on doors than the other three. One justification here is that there is no standard for heatmap reports in this kind of context. The same pixel that one tool marks as orange may be colored dark red by another, even though it shares the same attention probability in every prediction. This is a perfectly valid justification, and if these results could somehow be normalized or compared before visualizing the raw data, one could talk about consistency between different software [49].

Table 9 and Table 10 compare the area-of-interest values produced by 3M-VAS, EyeQuant, Attention Insight, and Expoze for daytime and nighttime views of the Military History Museum after the additional building was constructed. These comparative results demonstrate how different attention prediction algorithms identify points of perceptual attention in architectural structures. The availability of the polygonal option in the 3M-VAS and Expoze tools, especially during the drawing phase of areas of interest, makes these tools more preferable than others in architectural research. This is because architectural forms can take many different shapes. When comparing the findings, attention is paid to whether the areas of interest were created as rectangles or polygons. In this regard, when comparing the results of the AI-based visual attention software for the day and night views after the addition of the annex, some general trends as well as some differences are observed. As a general trend, the annex attracts less attention than the historical structure as a whole in all area of interest scores. When the results are considered, the tools with the highest overlap in the area of interest scores of the additional structure in the daytime and nighttime views are determined to be 3M VAS and Attention Insight, while the tool with the lowest overlap is determined to be EyeQuant.

## 5. Conclusions

The visual attention analyses performed with four artificial intelligence-based software indicate that the historic components of the Dresden Military History Museum generally attract more subconscious attention than the contemporary addition. This result confirms the hypothesis that historical elements systematically elicit greater pre-attentive focus than contemporary architectural additions at the Military History Museum. This study is limited to the case of the Military History Museum, and it is recommended that future research increase the number of cases to provide a more comprehensive and analytical view of the contemporary additions to historical structures.

Although the representations of heat maps and attention distribution diagrams obtained with four different AI-based software programs do not match exactly (e.g., color distribution and intensity in heat maps), they reveal that the new addition, despite having a contrasting character in terms of form and material, does not overshadow the historical structure. On the contrary, all outputs show that the modern addition creates a perceptible contrast that preserves the visual dominance and legibility of the original structure. These findings support the hypothesis that, although different AI-based visual attention analysis tools produce largely consistent results, a certain level of representational differences is inevitable.

Building on this, the study contributes to ongoing discussions about contrasting additions in historic environments by offering a quantifiable, visually based evaluation framework. Furthermore, it demonstrates how AI-based saliency visualization tools can be systematically used to compare visual attention in architectural settings. The combined use of heat maps, gaze sequence maps, and attention distribution diagrams establishes a reproducible visualization workflow that can support future architectural perception research.

The findings of the study indicate that, in the analyzed daytime views of the Military History Museum, the historical façade is predicted to receive higher visual saliency than the contemporary addition. While this outcome shows partial alignment with discussions within Cognitive Architecture Theory, it does not provide direct evidence of an inherent perceptual preference for traditional architectural forms. The present study did not ex-amine the influence of specific formal variables—such as symmetry, edge density, or luminance contrast—nor did it assess contextual factors including camera angle, back-ground composition, or the comparatively darker tonality of the contemporary intervention. Therefore, the observed distribution of visual saliency should be interpreted as specific to the particular visual configuration analyzed. Broader theoretical generalizations regarding traditional architecture or cognitive preference would require additional controlled analyses or comparative studies across multiple cases.

While artificial intelligence-based prediction tools do not fully replace real eye-tracking experiments due to the limitations of two-dimensional image analysis, they offer a practical and cost-effective method for preliminary visual assessments.

Overall, the study shows that visual attention software can serve as a useful analytical tool in architectural conservation debates, enabling a more objective understanding of how contrasting design approaches are visually perceived.

## Figures and Tables

**Table 1:** Artificial intelligence-based visual attention software.

Software	Company	Year, Location	Web Access Address
3M-VAS	3M Company	2010, Minneapolis, MN, USA	https://vas.3m.com, accessed on 10 October 2025
EyeQuant	WhiteMatter Labs GmbH	2013, Berlin, Germany	https://eyequant.com, accessed on 10 October 2025
Attention Insight	Attention Insight	2019, Hamburg, Germany	https://attentioninsight.com , accessed on 10 October 2025
Expoze	Alpha.One	2015, Amsterdam, Netherlands	https://expoze.app, accessed on 10 October 2025

**Table 2:** Visual data set used in the study.

Visual Type	Visual	Number of Images	Areas of Interest *
Historic Building (daytime)	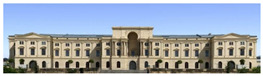	1	The monumental entrance facade of the symmetrical historic facade, left and right facades.
Contemporary Addition Historic Building (daytime-nighttime)	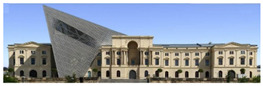	2	The facade of the add, right and left facades after the add.
	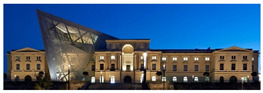		

* Depending on the type of AI-powered eye-tracking tool used, areas of interest can be drawn as rectangles or polygons.

**Table 3:** Visual attention analysis was conducted on day and night views of the Military History Museum both before and after the additional building was constructed using 3M-VAS.

3M-VAS results	The original state of the Military History Museum before the additional building	View of the Military History Museum after the additional building	Night view of the Military History Museum after the additional building
	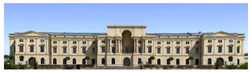	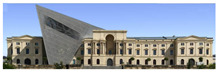	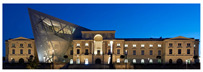
Heatmap	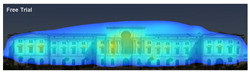	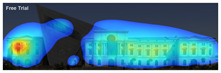	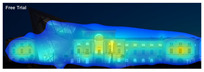
Hotspots	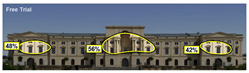	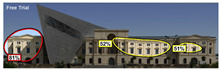	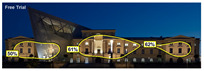
Gaze Sequence	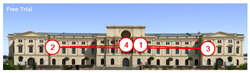	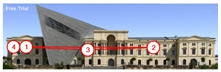	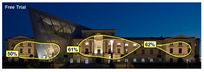
Visual Elements	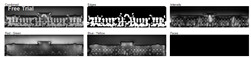	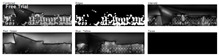	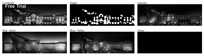
AOIs	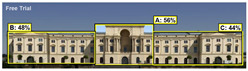	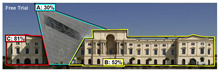	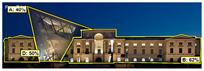
Visual Elements of AOIs	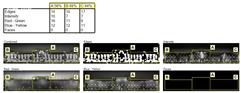	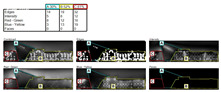	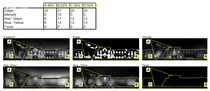

**Table 4:** Visual attention analysis was conducted on day and night views of the Military History Museum both before and after the additional building was constructed using Attention Insight.

**Attention Insight** **results**	The original state of the Military History Museum before the additional building	View of the Military History Museum after the additional building	Night view of the Military History Museum after the additional building
	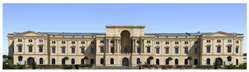	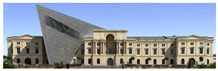	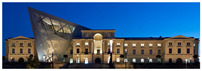
Attention Heatmap	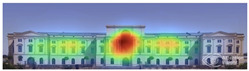	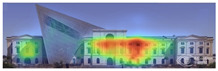	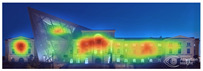
Focus Map	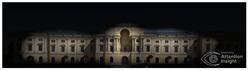	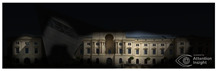	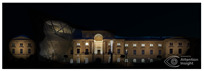
Contrast Map	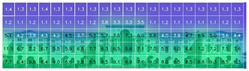	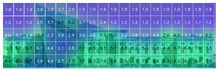	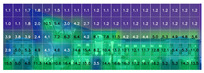
AOIs	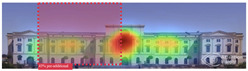	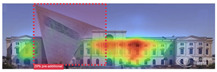	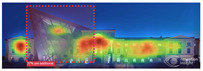
Clarity score	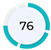	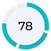	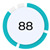
Focus score	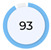	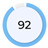	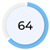

**Table 5:** Visual attention analysis was conducted on day and night views of the Military History Museum both before and after the additional building was constructed using EyeQuant.

EyeQuant results	The original state of the Military History Museum before the additional building	View of the Military History Museum after the additional building	Night view of the Military History Museum after the additional building
	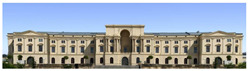	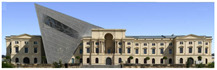	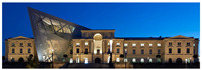
Heatmap/ Attention	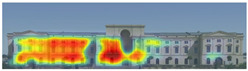	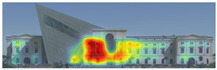	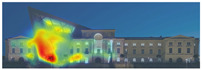
Perception Map	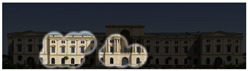	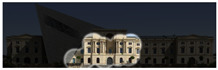	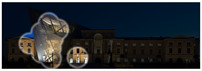
Hot spots	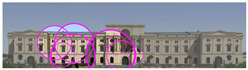	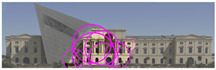	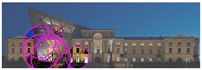
Clarity map	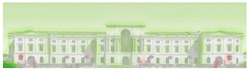	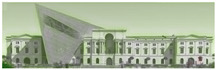	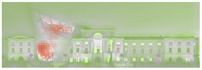
Clarity score	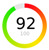	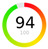	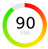
Excitingness map	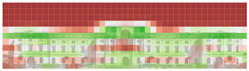	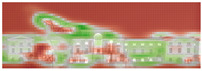	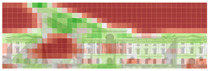
Excitingness score	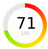	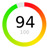	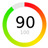
AOIs	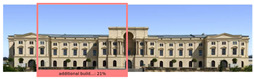	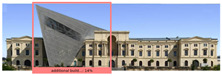	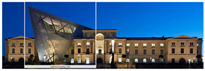

**Table 6:** Visual attention analysis was conducted on day and night views of the Military History Museum both before and after the additional building was constructed using Expoze.

Expoze results	The original state of the Military History Museum before the additional building	View of the Military History Museum after the additional building	Night view of the Military History Museum after the additional building
	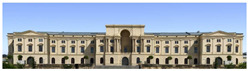	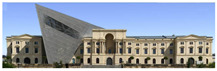	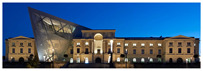
Heatmap	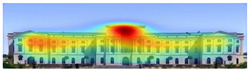	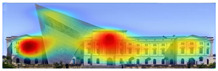	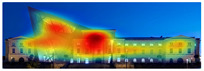
AOIs	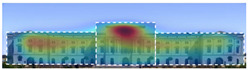	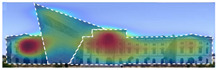	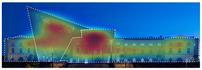
Values of AOIs	32.7%—**38.2%**—25.5%	15%—**21.9%**—55.4%	5.5%—**32.6%**—54.5%

**Table 7:** Heat map comparison of daytime views of the Military History Museum before and after the construction of the additional building.

Military History Museum Heat Map results	The original state of the Military History Museum before the additional building.	View of the Military History Museum after the additional building.
	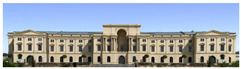	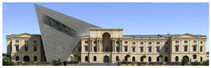
3M VAS	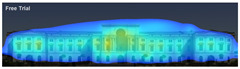	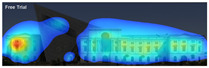
Attention Insight	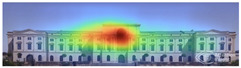	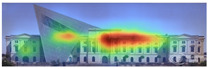
EyeQuant	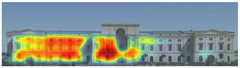	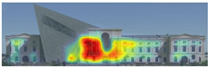
Expoze	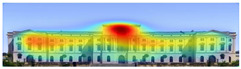	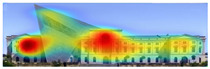

**Table 8:** Heat map comparison of daytime views of the Military History Museum before and after the construction of the additional building.

Military History Museum Heat Map results	Daytime view of the Military History Museum after the additional building.	Nighttime view of the Military History Museum after the additional building.
	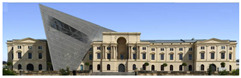	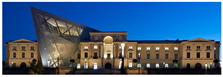
3M VAS	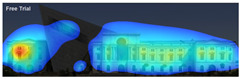	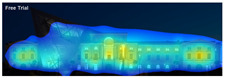
Attention Insight	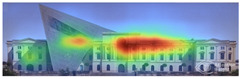	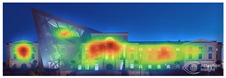
EyeQuant	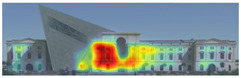	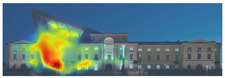
Expoze	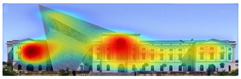	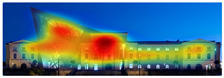

**Table 9:** Comparison of AI-based visual attention software interest area results, daytime view after additional construction.

Software	Analysis	AOIs Type	The AOI Score of the Additional Structure	The AOI Scores of the Historic Building Façade
3M VAS	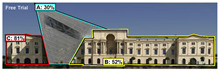	polygonal	30%	52-81%
Expoze	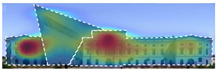	polygonal	21.9%	15–55.4%
Attention Insight	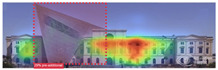	rectangle	29%	-
EyeQuant	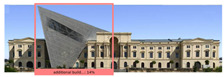	rectangle	14%	-

**Table 10:** Comparison of AI-based visual attention software interest area results, nighttime view after additional construction.

Software	Analysis	AOIs Type	The AOI Score of the Additional Structure	The AOI Scores of the Historic Building Façade
3M VAS	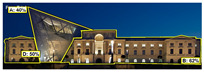	polygonal	40%	50—62%
Expoze	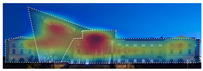	polygonal	32.6%	5.5—54.5%
Attention Insight	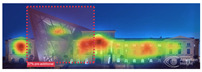	rectangle	37%	-
EyeQuant	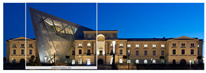	rectangle	48%	-

## Data Availability

The data presented in this study are available in the article.

## References

[1] Kurnaz A., Aniktar S. (2024). Visual Perception and contextual relationship of contemporary extensions and historical buildings. J. Archit. Conserv..

[2] Lavdas A.A., Salingaros N.A., Sussman A. (2021). Visual Attention Software: A New Tool for Understanding the “Subliminal” Experience of the Built Environment. Appl. Sci..

[3] Bojko A. (2013). Eye Tracking the User Experience: A Practical Guide to Research.

[4] Hollander J.B., Sussman A., Lowitt P., Angus N., Situ M. (2021). Eye tracking emulation software: A promising urban design tool. Archit. Sci. Rev..

[5] Ko T.K., Kim I.T., Choi A.S., Sung M. (2016). Simulation and perceptual evaluation of fashion shop lighting design with application of exhibition lighting techniques. Build. Simul..

[6] Jiang J., Meng Q., Yang D., Li M., Kang J. (2025). Simulating spatial experience in exhibition spaces with musical soundscapes using virtual reality. Build. Simul..

[7] Lavdas A.A. (2024). Eye-tracking applications in architecture and design. Encyclopedia.

[8] 3M-VAS Visual Attention Software. https://www.3m.com/3M/en_US/visual-attention-software-us/.

[9] Attention Insight. https://attentioninsight.com/.

[10] EyeQuant. https://www.eyequant.com/.

[11] Expoze. https://expoze.app/.

[12] Bente G. (2004). Erfassung und Analyse des Blickverhaltens. Lehrbuch der Medienpsychologie.

[13] Płuciennik M. (2018). The first hundred years: A history of eye tracking as a research method. Appl. Linguist. Pap..

[14] Jacob R.J., Karn K.S. (2003). Commentary on Section 4. Eye tracking in human-computer interaction and usability research: Ready to deliver the promises. Mind’s Eye.

[15] Tobii. https://www.tobii.com/company/this-is-tobii.

[16] Yuan L., Cao Z., Mao Y., Isa M.H.M., Abdul Nasir M.H. (2025). Age-Related Differences in Visual Attention to Heritage Tourism: An Eye-Tracking Study. J. Eye Mov. Res..

[17] Gazepoint. https://www.gazept.com/?v=e7d707a26e7f.

[18] Treisman A.M., Gelade G. (1980). A feature-integration theory of attention. Cogn. Psychol..

[19] Ariannia N., Naseri N., Yeganeh M. (2024). Cognitive-emotional feasibility of the effect of visual quality of building form on promoting the sense of place attachment (Case study: Cultural iconic buildings of Iran’s contemporary architecture). Front. Archit. Res..

[20] Xie Q., Zhang L. (2024). Entropy-based guidance and predictive modelling of pedestrians’ visual attention in urban environment. Build. Simul..

[21] Rusnak M.A., Rabiega M. (2021). The potential of using an eye tracker in architectural education: Three perspectives for ordinary users, students and lecturers. Buildings.

[22] Lavdas A.A., Salingaros N.A. (2022). Architectural beauty: Developing a measurable and objective scale. Challenges.

[23] Sherman K.D. (2019). Design Lost in Time: A Visual Attention Software (vas) Analysis of Historic Preservation in Da Nang, Vietnam and Boston, Massachusetts. Master’s Thesis.

[24] Higuera Trujillo J., Marín Morales J., Rojas J., López Tarruella Maldonado J. Emotional maps: Neuro architecture and design applications. Proceedings of the Systems & Design: Beyond Processes and Thinking.

[25] iMotions. https://imotions.com/blog/insights/trend/future-eye-tracking-technology/.

[26] Erkartal P.Ö., Özüer M.O. Imitating or Ignoring the Historical Texture?. Proceedings of the Fill in the Blanks, Fener-Balat Workshop.

[27] Hossein Askari A., Dola K., Soltani S. (2014). An evaluation of the elements and characteristics of historical building façades in the context of Malaysia. Urban Des. Int..

[28] Silveira da Costa V., Montagna da Silveira A., da Silva Torres A. (2022). Evaluation of degradation state of historic building facades through qualitative and quantitative indicators: Case study in Pelotas, Brazil. Int. J. Archit. Herit..

[29] Tabrizi S.K., Abdelmonem M.G. (2024). Contemporary construction in historical sites: The missing factors. Front. Archit. Res..

[30] Demir Ç. (2021). Harmonical Contrast Design Approach in Historical Urban Context. Master’s Thesis.

[31] Li X., Zhang Y. (2023). Conserving and managing historical urban landscape an integrated morphological approach. European Planning Studies.

[32] Ching F.D. (2023). Architecture: Form, Space, and Order.

[33] Zhu Y., González Martínez P. (2022). Heritage, values and gentrification: The redevelopment of historic areas in China. Int. J. Herit. Stud..

[34] Pronina T.V. (2021). The Method of Contrast of Modern Architecture in the Historical Environment of the City. Proceedings of the International Scientific Conference “Construction and Architecture: Theory and Practice of Innovative Development (CATPID 2020)”.

[35] Munasinghe H. (2022). Proclaiming Colonial Urban Heritage: Towards an Inclusive Heritage-interpretation for Colombo’s Past. J. Contemp. Urban Aff..

[36] Uğur Ö.T. (2020). Contemporary Additions to Historic Buildings: Parasite Structures. Arkitekt.

[37] Salingaros N.A., Sussman A. (2020). Biometric pilot-studies reveal the arrangement and shape of windows on a traditional façade to be implicitly “engaging”, whereas contemporary façades are not. Urban Sci..

[38] Gökaslan A., Erkan İ. (2020). A cognitive investigation of interior effects of window sizes. New Des. Ideas.

[39] Azzam Z.N., Al-Moqaram A.M. (2024). Simulating eye tracking in buildings facades to understand the impact of visual experience and sensory responses: University of Baghdad facades as a case study. AIP Conf. Proc..

[40] Türken A.Ö. Examining the Impacts of Golden Horn Metro Bridge on Historical Skyline Through Visual Attention. Proceedings of the CPUD ’22, VII International City Planning and Urban Design Conference.

[41] Salama A.M., Salingaros N.A., MacLean L. (2023). A multimodal appraisal of Zaha Hadid’s Glasgow Riverside Museum—Criticism, performance evaluation, and habitability. Buildings.

[42] Mafi N. 14 Beautiful Examples of When Historic and Modern Architecture Come Together. https://hsi-eg.com/14-Beautiful-Examples-of-When-Historic-and-Modern-Architecture-Come.

[43] Ascher Barnstone D. (2020). Paradoxes of war critique on display: The Dresden Bundeswehr Museum of Military History. Aust. New Zealand J. Art.

[44] Yazman D. Libeskind’s Glass Partition Pierces the Dresden Military Museum. http://www.arkitera.com/haber/libeskindin-cam-bolmesi-dresden-askeri-muzesini-delip-geciyor/.

[45] Studio Libeskind. https://libeskind.com/work/military-history-museum.

[46] Rusnak M., Szewczyk J. (2018). Eye trackers in evaluation of transformation of historical monuments. Revitalisation of the Dresden arsenal. E3S Web Conf..

[47] De la Fuente Suárez L.A. (2020). Subjective experience and visual attention to a historic building: A real-world eye-tracking study. Front. Archit. Res..

[48] Hollander J.B., Sussman A., Lowitt P., Angus N., Situ M., Magnuson A. (2022). Insights into wayfinding: Urban design exploration through the use of algorithmic eye-tracking software. J. Urban Des..

[49] Vitruvius Grind Angels in the Architecture (Interlude). https://thehustlearchitect.substack.com/p/angels-in-the-architecture-interlude.

